# Spatial shaping of neural activity using electrical stimulation

**DOI:** 10.1186/1471-2202-14-S1-P49

**Published:** 2013-07-08

**Authors:** Hamish Meffin, Bahman Tahayori, Elma O'Sullivan Greeene, David B Grayden, Anthony N Burkitt

**Affiliations:** 1 Victorian Research Laboratory, National ICT Australia, Carlton, Victoria 3010, Australia; 2 Neural Engineering Laboratory, Department of Electrical & Electronic Engineering, The University of Melbourne, Carlton, Victoria 3010, Australia; 3 Centre for Neural Engineering Laboratory, The University of Melbourne, Carlton, Victoria 3010, Australia

## 

Neuroprostheses such as cochlear, retinal or cortical implants are used to restore lost function to specific parts of the nervous system in an increasing variety of patients. These prostheses work by electrically stimulating a group of neurons that have impaired or lost function, using an implanted array of electrodes.

A major problem with using electrical current to stimulate neurons is that the diffuse spread of current through the neural tissue limits the resolution of these devices. If electrodes on the array are placed closer together than the distance of current-spread, then combined stimulation will result in a blurred aggregate of neural activity.

We present a theoretical framework for spatially shaping neural activity that overcomes this limitation of electrical stimulation. The approach relies on applying a combination of positive and negative currents across the electrodes of the array, in such a way that the aggregate neural activity is reinforced at desired locations, but cancelled at other locations. By choosing the current amplitudes on the electrodes to be sufficiently large (i.e. increasing the gain), high resolution can be recovered: resolution that is limited by the electrode spacing rather than the current-spread.

To this end we present a mean-field formalism for calculating spatio-temporal patterns of neural activity evoked by an array of electrodes. The formalism allows the calculation of the extracellular, intracellular and subthreshold membrane potential, as well as mean spike rate.

The formalism allows modeling at macro- and micro- scales to be performed self-consistently, so that the electrical impedance of a volume of tissue is matched to the electrical impedance of a single neurite in a mean-field approximation. This allows consistent modeling across the necessary spatial scales of the problem: from current flow between electrodes through tissue, to the the stimulation of individual neurites embedded in that tissue. This self-consistency is critical to avoid ambiguities that lead to non-unique (i.e. contradictory) model predictions.

The formalism describes two relevant modes of stimulation: a longitudinal mode, described by a classical cable equation, and a transverse mode, described by a novel ordinary differential equation. These modes are illustrated and their magnitudes compared as a function of electrode-neurite separation and stimulus pulse duration.

Finally we show how the model is utilized to shape evoked neural activity to approximate an arbitrary target spatial pattern of neural activity. This involves a global optimization over the current amplitudes on the electrodes to minimise the mean square difference between the model's predicted pattern of activity and the target pattern. Simulations using the example of a model of retinal stimulation show large improvements in resolution are possible, so that resolution is limited by electrode spacing rather than the current spread from an electrode.

In order to permit the possibility of real time implementation for clinical application, the algorithm can be split into linear and non-linear stages. Computational speed is improved by several orders of magnitude by pre-computing the linear spatial maps arising from each electrode individually, which can then be used to predict the activity resulting from combined stimulation by a superposition principle.

